# Climate change and sustainable healthcare practices in nursing: A multi-country exploratory online survey

**DOI:** 10.1016/j.joclim.2026.100656

**Published:** 2026-04-16

**Authors:** Ebenezer Akore Yeboah, Amanda Rodrigues Amorim Adegboye, Laura Wilde, Om Kurmi, Rosie Kneafsey

**Affiliations:** Research Centre for Healthcare and Communities, Coventry University, UK

**Keywords:** Climate change, Nursing, sustainability, Environmentally responsible healthcare, Net-zero healthcare, Sustainable healthcare practices

## Abstract

**Introduction:**

Healthcare systems are responding to the immediate and long-term impacts of climate change by providing care, and implementing carbon reduction initiatives. The nursing profession possesses substantial potential to advocate and embody sustainability values. However, a proportion of nurses remain unaware of the intricate linkages between nursing practices and climate change; hence this study explored nurses’ and midwives’ awareness, perceptions and attitudes regarding climate change and sustainable healthcare practices.

**Methods:**

A cross-sectional online survey using a mixed-methods approach was conducted, targeting registered nurses and midwives. Utilising a convenience sampling technique, a total of 473 participants from 56 countries completed a self-administered survey comprising both closed and open-ended questions. Descriptive analysis was used for the quantitative data and thematic analysis was conducted with the qualitative data.

**Results:**

The majority (86%) of respondents reported awareness of anthropogenic causes of climate change; however, only 33% were aware of the term ‘net-zero healthcare’. Common barriers noted were inadequate implementation of environmental policies (76.9%), work overload (72.2%), lack of organisational support (60.3%), and less time to think about environmental impact (55.8%). Three main themes were identified from the qualitative data, including (i) sources of healthcare carbon footprint, (ii) actions towards net-zero healthcare, and (iii) factors influencing the nursing role in environmental sustainability.

**Conclusion:**

The findings highlight inadequacies in healthcare's climate action efforts, emphasizing the need for healthcare organizations to evaluate their climate policy awareness. Integrating climate change and sustainability into nursing curricula and embedding carbon reduction policies within healthcare organizations are essential for improving climate action.

## 1 Introduction

Climate change poses a significant threat to the growth and survival of humanity. In scientific discourse, climate change refers to alterations in the climate caused by both human activities and natural events or solely by anthropogenic influences [1]. The prevailing global scientific consensus, as stated in the Geneva Accord of 2013, attributes climate change primarily to human activities. This attribution is supported by numerous instances of ‘human fingerprints’ identified through the measurement of carbon isotopes [2,3]. The impacts of climate change extend beyond immediate human suffering, contributing to environmental degradation and hindering economic development. These impacts hinder human development by diverting funds from growth-oriented projects to disaster recovery and mitigation, thereby impeding overall progress and prosperity [4,5].

As healthcare systems globally work towards the net-zero agenda, nurses and midwives play a part in reducing their footprint, since they use a wide variety of care products which have a carbon footprint [6]. For example, nurses frequently use wound dressing packs, which may include unused items [7]. Nurses may also be responsible for prescribing treatments, such as inhalers and other medications, ordering consumables, or commissioning services. Underpinning procurement and commissioning processes with a carbon reduction remit enables healthcare settings to identify unnecessary items or adjust purchasing in quantities to minimize waste, and reduce the overall carbon footprint of the healthcare system. One critical source of carbon arises from healthcare-associated transport. A case study in Central Manchester University Hospitals, UK, reported that nurses contributed most to the hospital’s transportation carbon emissions [8]. As the largest workforce group, this is unsurprising, but points to the need to recognise the potential impact on key groups (both positive and negative), when sustainable practices such as shared transportation are encouraged [9].

In relation to climate change awareness, a study conducted in Rwanda with 184 nurses and midwives demonstrated that 60.4% had a low awareness level of climate change and its impacts on neonatal health [10]. Moreover, in a study conducted within the UK National Health Service (NHS), nurses were largely unaware of the hospital’s sustainability initiatives related to transportation [8]. These findings highlight a critical gap in communication and raise concerns about the effectiveness of healthcare organisations in informing and engaging their staff on climate change policies and related initiatives. While some nurses acknowledge a connection between their work and climate change, particularly in terms of treating and caring for individuals affected by climate-related health issues, recognition of the broader contribution of healthcare and nursing practices to carbon emissions remains limited [11,12]. It is therefore important to explore nurses’ and midwives’ awareness, perceptions and attitudes in relation to climate change and sustainable healthcare practices in many settings. While this study employs a multi-country design to capture a breadth of perspectives, its primary goal is exploratory, mapping shared and divergent themes across nursing contexts rather than asserting representative conclusions.

## 2 Methods

### 2.1 Study design

A mixed-method, cross-sectional online survey design was used. The cross-sectional approach prioritises initial insights over generalisability, laying the groundwork for future targeted research.

### 2.2 Study population and recruitment strategy

The study population included registered nurses or midwives globally irrespective of workplace or setting. The survey was promoted on the Nursing Now Challenge (NNC) website in their news section [13]. The survey link was also disseminated via the Chief Nurses Bulletin at a local hospital in the UK, and the UK Florence Nightingale Foundation. Further distribution occurred via multiple social media platforms such as Facebook, Twitter, and the World Health Organisation (WHO) network. Reminders and reposting were completed twice on social media during the data collection period from 5th June 2023 to 6th November 2023.

### 2.3 Sampling technique

As a global survey, internet-based data collection was chosen to facilitate easy access to diverse and geographically dispersed people [14,15].

### 2.4 Sample Size

A priori power analysis was completed using G*Power and an assumed attrition rate of 20% revealed an estimate of 250 participants. The research team assumed a moderate effect size of 0.03 and an acceptable power of 80%. The significance level was set at 5%. The model followed a fixed model, single regression coefficient, two-tailed tests with 39 predictors from the independent variables and a degree of freedom of 168.

### 2.5 Eligibility

The study included qualified nurses and midwives regardless of work setting or country of practice. This study concentrated on registered professional nurses and midwives because student nurses are less likely to have access to hospital intranet information regarding policies and training.

### 2.6 Data collection

All the participants had access to the Qualtrics survey link to voluntarily participate by completing a consent form before proceeding to the questions. The online self-completed survey had both closed and open-ended questions. While the survey included open-ended questions, this qualitative component was not intended to fulfil the depth of a standalone qualitative study. Instead, it complemented the quantitative data by capturing emergent themes from nurses’ perspectives. The closed-ended questions utilised a mixture of three and four Likert scales [16,17] in order to reduce the risk of satisficing behaviour and counterbalance the risk of forced-response [17,18]. The survey included a demographic section with six questions, eight sets of closed-ended questions on climate change awareness, attitude towards climate change, awareness of climate policies, workplace mandatory training, organisational values, perception of climate change, range of sustainable healthcare practice and use of Personal Protective Equipment. The survey also included five open-ended questions which included thoughts on harmful environmental practices, nursing autonomy and nursing influence on climate policy (See Appendix, survey questionnaire).

The survey questions were derived from the research questions and informed by relevant literature. Their development was further guided by findings from a mixed-methods systematic literature review conducted by the researchers [12]. Some survey questions related to nursing and/or climate change from other studies [18,19,30] and from grey literature sources [[20], [21], [22], [23]] were utilised in the tool development. A pilot test of the survey was conducted with a total of 10 nurses. The sample included five registered nurses employed in teaching roles in the School of Nursing, Midwifery and Health (UK) and five registered nurses working clinically in a local university hospital. These were purposely sampled to represent views from academics, healthcare leaders and clinicians. Feedback from two respondents were used to refine the questions before the final survey was launched. A reliability test of the piloted results was performed to assess the consistency and precision of the statistical analysis. Cronbach’s alpha of 0.802 was noted, indicating that the measure is highly reliable.

### 2.7 Data Analysis

The quantitative data was exported from Qualtrics into SPSS version 28 for processing and analysis [24]. Descriptive statistics were used to analyse the quantitative data. We conducted additional statistical comparison (e.g. ANOVA) to examine regional differences in key variables such as awareness, perception, and attitude (See supplementary file, Table 1). The qualitative data were exported into NVIVO software [25] which underwent thematic analysis by following Braun and Clark's six steps [26] (See supplementary file, Table 2). The relevant data extracts used in the final reporting were pseudo-identified. The data analysis process followed a semantic and inductive approach. Themes were iteratively refined through focused discussions among the research team for approximately 6 times, review of the written analysis was done approximately 9 times, and representative quotes were selected to ensure confidence in the analysis. In this paper, ‘extremely’ and ‘moderately’ are grouped to reflect the general direction of the response rather than the degree or intensity of the measurement.

**Table 1:** Socio-demographic characteristics of participants.

**Characteristics of participants**	**Frequency (n)**	**Percentage (%)**
**Gender (*n* = 373)**		
Men	62	16.6%
Women	292	78.3%
Others	12	3.2%
Prefer not to say	7	1.9%
**Age (years) (*n* = 372)**		
40 or less years	211	56.7%
More than 40 years	161	43.3%
**Educational level (*n* = 471)**		
Undergraduate	297	63.0%
Postgraduate degree	174	37.0%
**Years of work experience (*n* = 368)**		
5 years or less	120	32.6%
More than 5 years	248	67.4%
**Level of seniority (*n* = 472)**		
Junior	97	20.6%
Middle	147	31.1%
Senior	184	39.0%
Consultant	18	3.8%
Other	26	5.5%

Missing data: education (*n* = 2), level of seniority (*n* = 1), gender (*n* = 100), age (*n* = 101), work experience (*n* = 105).

**Table 2:** Distribution of country of practice of participants.

**Countries by continents**	**Number of respondents (*n* = 470)**	**Percentage**
**Fifteen countries represented Africa.** Cameroon, Egypt, Ethiopia, Gambia, Ghana, Kenya, Liberia, Mauritius, Nigeria, Rwanda, South Africa, Sudan, Tanzania, Uganda, Zambia	140	29.8%
**Six countries represented North America** Barbados, Belize, Canada, Panama, St. Kitts and Nevis, USA	83	17.7%
**Three countries represented South America** Argentina, Brazil, Guyana	8	1.7%
**Eighteen countries represented Europe.** Austria, Belgium, Croatia, Cyprus, Denmark, Germany, Ireland, Isle of Man, Italy, Latvia, Malta, Netherlands, Portugal, Romania, Spain, Switzerland, Ukraine, United Kingdom	193	41.1%
**Twelve countries represented Asia** Bangladesh, Bhutan, India, Indonesia, Korea, Nepal, Philippines, Saudi Arabia, Singapore, Syria, Taiwan, Turkey	37	7.9%
**Two countries represented Oceania/Australia** Australia, New Zealand	9	1.9%

Missing data: country of practice (*n* = 3)

## 3 Results

The total number of respondents used in the analysis was 473, from 56 countries across 6 continents, though not all participants had complete data.

### 3.1 Demographics features

The respondents primarily identified as women (78.3%), 40 years of age or less (56.7%), and had an undergraduate degree (63.0%). The majority of respondents had more than five years of work experience (67.4%) and were in a senior role (39.0%) (See Table 1).

Missing data: education (*n* = 2), level of seniority (*n* = 1), gender (*n* = 100), age (*n* = 101), work experience (*n* = 105). Participants were also asked to subjectively place themselves in their respective levels of seniority.

One hundred and ninety-three respondents (193) were from 18 European countries, followed by 140 from 15 African countries, 37 from 12 Asian countries, and 11 from other continents (see Table 2).

### 3.2 Distribution of awareness of climate change

**Fig. 1:**
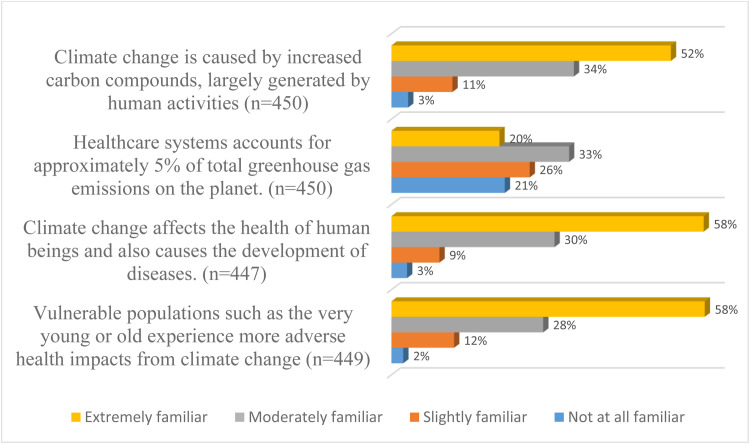
Distribution of awareness of climate change.

About 86% of the respondents reported moderate or extreme awareness of human activity as a major cause of climate change, and 88% identified that climate change does affect human health and causes disease development. However, only 53% were aware of the percentage of the total global carbon footprint contributed by healthcare systems (see Fig. 1).

### 3.3 Distribution of attitude towards climate change

**Fig. 2:**
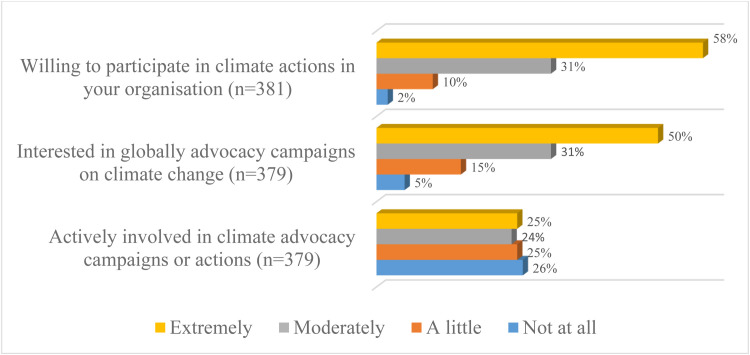
Distribution of attitude towards climate change.

Approximately nine in ten participants (89%) indicated they were moderately or extremely willing to participate in climate actions in their local organisation while 81% were interested in global climate advocacy campaigns. However, only half of the respondents (49%) were actively involved in these campaigns (see Fig. 2).

### 3.4 Distribution of perception on climate change and nursing

**Fig. 3:**
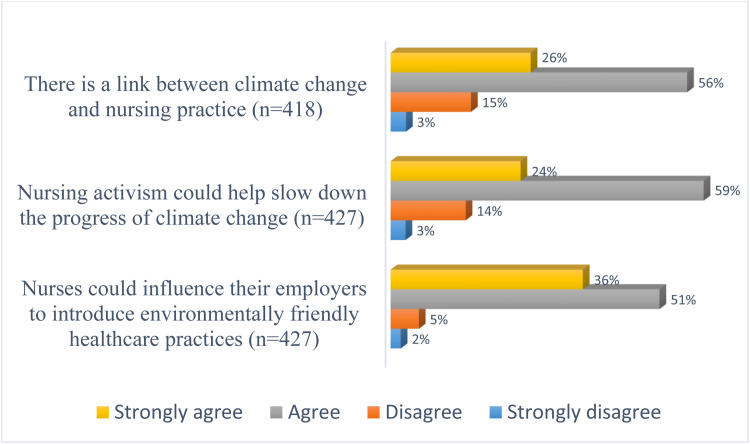
Distribution of perception on climate change and nursing.

About 82% of participants agreed that there is a connection between nursing practice and climate change while 83% agreed that nursing activism could help reduce the progress of climate change. Also, 87% reported that nurses could influence their employers in the introduction of environmentally friendly practices (see Fig. 3).

### 3.5 Distribution of awareness of organisational or institutional climate policies

**Fig. 4:**
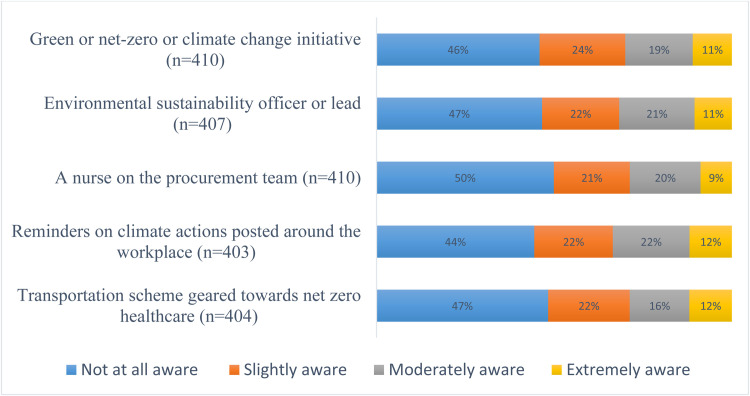
Distribution of organisational or institutional climate policies.

**Fig. 5:**
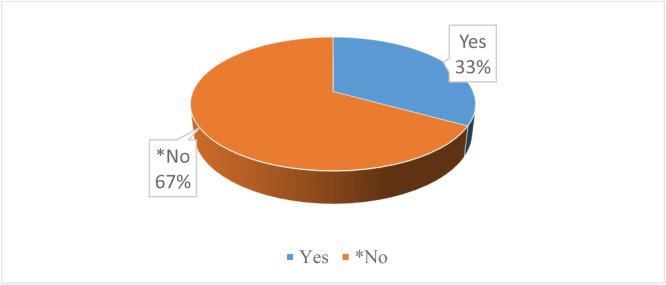
Distribution of awareness of net-zero healthcare.

The findings reveal limited awareness of net-zero ('green') initiatives among respondents, with approximately seven out of 10 reporting slight or no awareness. Similarly, 69% indicated minimal or no familiarity with the role of an environmental sustainability officer or transportation schemes aligned with net-zero goals. Half of the respondents were unaware of nurse representation on their institution’s procurement team. Additionally, 66% reported slight or no exposure to climate action reminders, while 67% were unfamiliar with the term ‘net-zero healthcare’ (see Figs. 4 & 5).

### 3.6 Distribution of participants’ experience on workplace training on climate change and nursing

**Fig. 6:**
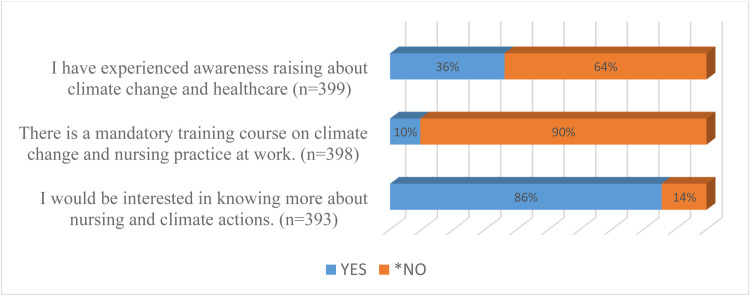
Distribution of workplace mandatory training of climate change.

The majority (90%) of respondents indicated that there was no mandatory training on climate change and nursing practice in their workplace. Additionally, 64% reported no awareness-raising at work. However, 86% would be interested to know more about climate change and nursing (see Fig. 6).

### 3.7 Distribution of participants’ environmentally friendly healthcare practices

**Fig. 7:**
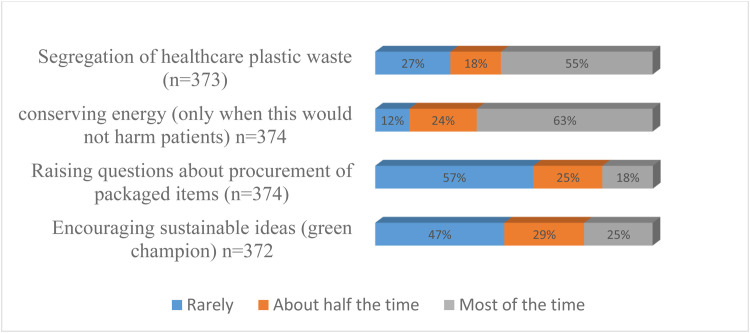
Distribution of environmentally responsible healthcare practices.

A little over half (55%) of the respondents reported that they segregate healthcare waste, though 27% reported rarely doing this. Also, 87% of respondents indicated they conserved electrical energy when they felt it would not harm patients. Conversely, 57% of the respondents rarely raised questions on procurement and 47% rarely encouraged sustainable ideas or green champions (see Fig. 7).

### 3.8 Barriers to practicing environmentally responsible healthcare

Inadequate implementation of environmental policies, work overload, lack of organisational support, and less time to think of the environment during patient care were the common barriers reported (see Table 3). Multiple responses were allowed and results are presented in rank order of the total percent of cases.

**Table 3:** Barriers to practicing environmentally friendly healthcare.

**Main barriers to practicing environmentally friendly healthcare**	**Frequency (*n* = 386)**	**Percentage (%)**
Inadequate implementation of environmental policies	296	76.9%
Busy shift/ work overload	278	72.2%
Lack of organisation support	232	60.3%
Less time to think of the environment during patient care	215	55.8%
Lack of autonomy in decision making	136	35.3%
Unnecessary procurement	107	27.8%
I do not know enough about climate change	100	26.0%
I do not know what to do	77	20.0%
Fear of getting infections	72	18.7%
I believed my role should only focused on patient need	62	16.1%
My climate change actions have very little to offer	55	14.3%
It is not my duty to reduce the carbon footprint of my workplace	33	8.6%

### 3.9 Thematic analysis findings

356 out of 473 (75%) participants responded to at least one of the five open-ended questions on the role of nursing in sustainability. Three main themes were identified; (i) sources of healthcare carbon footprint, (ii) actions towards net-zero healthcare, and (iii) factors influencing the nursing role in environmental sustainability.

#### 3.9.1 Theme 1: Sources of healthcare carbon footprint

Single use items, such as gowns, blood pressure cuffs or single use pulse oximeters, and improper waste segregation were identified as common sources of carbon footprint.

‘Single use gowns, overuse of gloves, unnecessary cannulation’ [Global North (Europe) respondent, I.D #37]

‘Any use of single use plastic, dialysis, washing, medication, single use NG (nsasogastric) syringes, lights, lots of equipment, ventilators’ [Global North (Europe) respondent, I.D #102]

‘Lack of proper waste segregation after nursing procedures hence causing inappropriate disposal of the waste products’ [Global South (Africa) respondent, I.D #128]

Packaging of healthcare items was reported as a major sources of plastic wastes.

‘Patients' drugs could be packaged in paper instead of plastics’ [Global South (Africa) respondent I.D #22]

‘The amount of waste we have after opening multiple supplies and plastic containers throughout just one shift’ [Global North (North America) respondent, I.D #368]

#### 3.9.2 Theme 2: Actions towards net-zero healthcare

Respondents reported that proper waste segregation systems, reducing paper use, returning to re-use models and employing green procurement are important approaches toward reducing the healthcare carbon footprint. It was also disclosed by participants that educational trainings could promote awareness of climate actions among health workers and the public.

‘Continuous sensitization of the public on the need to protect the environment. Also, health care workers should be made aware of the effects of our actions’ [Global South (Africa) respondent, I.D #46]

‘Climate change should be addressed in nursing curriculum at the first year of training and taught throughout the training periods so the environmentally friendly mind-set can be cultivated early enough… [AND] we can implement mandatory yearly or quarterly modules…’ [Global South (Africa) respondent, I.D #195]

Inclusion of net-zero evaluations in the assessment of hospital performance was suggested as potentially important performance metrics in the fight against climate change.

‘…Our regulatory bodies have performance measures that each hospital is held accountable to. If there is a desire …climate change should be added to the performance improvement metrics enforced by the existing regulatory bodies’ [Global North (North America) respondents, I.D #295]

Containerisation of waste and good waste segregation identifiers are key to reducing carbon footprint.

‘Adequate waste bins should be provided, coded, and well labelled to enhance waste segregation’ [Global South (Africa) respondent, I.D. 28]

‘Yes by learning to recycle and only using PPE when necessary and making use of the colour coding for waste disposal’ [Global South (Africa) respondent, I.D. 11]

#### 3.9.3 Theme 3: Factors influencing the nursing role in environmental sustainability

The sense of moral duty to keep the planet for future generations was reported as a motivating factor for nurses and midwives to deliver climate-oriented actions in their practices.

‘Yes, because I find that confronting climate change starts with each individual… it is our duty to protect our planet…bring awareness to my entire work on environment’ [Global South (Asia) respondent, I.D #132]

‘Absolutely, I feel we have a moral duty to protect our environment and minimize the health consequences of climate change and environmental pollution and stressors’ [Global North (Europe) respondent, I.D 86]

Conversely, nurses cited dilemmas in relation to infection prevention and control (IPC) standards, and workforce crises which sometimes contradicted their desire to be environmentally sustainable. One respondent stated;

‘Our standards are not in line with the risk assessment policy and as such we fail accreditation for not wearing PPE even when it is not indicated’ [Global North (Europe) respondent, I.D #102]

‘Feel like we have more pressing issues at my workplace such as staffing’ [Global North (North America) respondents, I.D #390]

‘No. Procurement & IPC are so heavily institutionalized that change is very difficult’ [Global North (North America) respondents, I.D #191]

## 4 Discussion

This research study provided important insights into nurses’ awareness, perceptions and practices related to environmentally sustainable healthcare in their workplaces. While our findings capture perspectives across multiple countries, the uneven distribution of participants means that these results should be interpreted with caution and viewed as identifying potentially important trends for future research rather than as definitive cross-national comparisons. A significant gap in climate policy awareness was evident amongst respondents, with approximately 70% indicating they were unfamiliar with the concept of ‘net-zero healthcare.’ This limited awareness suggests that healthcare organisations are not yet fully leveraging their potential to contribute positively to climate change mitigation. Notwithstanding, approximately 30% of respondents reported awareness of transportation schemes within their institutions. This result contrasts the findings of Nieto-Cerezo [8], which reported no awareness of transportation-related sustainability initiatives. These discrepancies may indicate variability in the implementation and communication of climate policies across different healthcare organisations and the time difference in both studies [27,28].

Contrary to the study by Anaker et al. [29] which reported comprehension of local climate actions, our study reported that high numbers of respondents (89%) were willing to participate in climate actions both locally and internationally which could be a platform for a nursing response to the unfolding climate crisis. This positive attitude could be an advantage in creating desire and drive toward environmentally responsible healthcare practices. Also, our study found a perceptible connection between nursing practice and climate change, which is contrary to previous studies [11,30], which reported no recognition of such link. This acknowledgement suggests a growing awareness among nursing professionals of their potential role in mitigating climate change through their practice [31]. Nursing activism encompassing individual actions and broader advocacy for systemic changes within healthcare organisations could contribute to the development of sustainable care models and collaborations [32,33].

Conversely, the common notion that infection prevention and control (IPC) is an impedance to sustainability [34] ranked ninth in this study. Sustainability leads and IPC leads could work closely and develop guiding policies and ensure its implementation for best practices. Responsible procurement, technology, and educational trainings are probable means of promoting climate actions among nurses and healthcare professionals. Building on our findings, environmental sustainability could be integrated into undergraduate and postgraduate nurse education globally. New programmes and recommendations can be developed by policymakers to ‘green’ healthcare systems globally. Additionally, the research suggests the need for an organisational assessment tool to measure sustainable practices and their awareness among healthcare workers [31]. Furthermore, the findings can support the implementation of carbon literacy training within hospitals.

### 4.1 Study limitation and strengths

We acknowledge some limitations in this study. Firstly, participant representation was uneven across countries, potentially introducing geographic bias. Additionally, missing data were observed, and small subgroup sizes in underrepresented regions may have limited statistical power. While convenience sampling was pragmatic for this first cross-national exploration of nursing and climate change, future studies would benefit from more balanced representation through alternative sampling strategies (e.g., multi-stage or random sampling), particularly in underrepresented regions. Furthermore, because the study was conducted in English, participation may have been restricted in non-English-speaking countries. Given these constraints, the findings should be interpreted with caution and viewed as exploratory trends rather than as globally representative. Consequently, the results may not be generalizable to the broader nursing population.

Despite these limitations, this study is the first mixed-methods, multi-country online survey exploring climate change and sustainable healthcare practices in nursing. The topic area is understudied and this study leads to knowledge exposure and individual assessment within the nursing field, and lays the groundwork for further investigation and dialogue on sustainability in healthcare. The trustworthiness of this study was enhanced by embedding Lincoln and Guba’s four criteria; credibility, transferability, dependability and confirmability [35]. Credibility was supported through triangulation of quantitative and qualitative data, iterative team discussions on emerging themes, and the presentation of representative verbatim quotes. Transferability was enhanced by providing detailed demographic characteristics and contextual information regarding healthcare settings. Dependability was promoted through a documented audit trail of survey development, piloting, coding procedures, and use of NVIVO and SPSS software. Confirmability was supported by linking interpretations to participant narratives and maintaining reflexive discussions among the research team to minimise researcher bias.

## 5 Conclusion

While awareness of climate change itself appears to be increasing, there remains a critical need to improve the dissemination and implementation of specific sustainability policies within healthcare organisations, particularly to ensure alignment with global climate action goals. Entrenched workplace cultures and inadequate policy implementation remain major barriers, highlighting the need for stronger organisational leadership to support sustainability initiatives. Given the urgency of the climate crisis, integrating climate change competencies and carbon literacy training into nursing education is recommended, alongside targeted research in underrepresented regions to better understand contextual barriers and test effective interventions.

## Funding statement

This PhD study is funded by the Research Centre for Healthcare and Communities, Coventry University, UK.

## Ethics/ Consent

Ethical approval (P146591) was granted by the Ethics Committee, Coventry University, UK. Participation was voluntary. Participants had to complete the consent form before participating. All participants were anonymised and all data received during the project were stored on a Coventry University encrypted laptop and Coventry University Office 365, including OneDrive and Stream.

## Patient and public involvement

Patients and/or the public were not involved in the design, or conduct, or reporting, or dissemination plans of this research.

## Patient consent for publication

Not applicable.

## Data sharing statement

The raw or processed data required to reproduce the above findings cannot be shared at this time as the data also forms part of an ongoing PhD study, however, the data will become available from the corresponding author [EAY] on request after PhD is completed or thesis submitted in 2026.

## CRediT authorship contribution statement

**Ebenezer Akore Yeboah:** Writing – review & editing, Writing – original draft, Visualization, Validation, Software, Methodology, Investigation, Formal analysis, Data curation, Conceptualization. **Amanda Rodrigues Amorim Adegboye:** Writing – review & editing, Writing – original draft, Validation, Supervision, Software, Methodology, Funding acquisition, Formal analysis, Conceptualization. **Laura Wilde:** Writing – review & editing, Writing – original draft, Validation, Supervision, Methodology. **Om Kurmi:** Writing – review & editing, Writing – original draft, Validation, Supervision, Methodology. **Rosie Kneafsey:** Writing – review & editing, Writing – original draft, Validation, Supervision, Methodology, Investigation, Funding acquisition, Formal analysis, Conceptualization.

## Declaration of competing interest

The authors declare that they have no known competing financial interests or personal relationships that could have appeared to influence the work reported in this paper.

## Appendix Supplementary materials

Image, application 1

Image, application 2

Image, application 3

## Footnotes

Supplementary material associated with this article can be found, in the online version, at doi:10.1016/j.joclim.2026.100656.
