# Supplementary material for: Climate change and sustainable healthcare practices in nursing: A multi-country exploratory online survey
Source: J Clim Chang Health. 2026 Apr 16;29:100656. doi: 10.1016/j.joclim.2026.100656 (PMC13101775; doi:10.1016/j.joclim.2026.100656)
Supplement: Supplementary file 2 [file mmc2.docx]

**Supplementary file**

**Distribution of one-way analysis of variance among variables**

A series of one-way ANOVA was performed to understand whether climate change awareness, perception, attitude, climate policy awareness, and practices. There was statistically significant differences between continents and the outcomes. P< 0.05

Table 1: series of one-way ANOVA

|  | Sum of squares | Df | Mean square | F | Sig. |
| --- | --- | --- | --- | --- | --- |
| Awareness: There was a statistically significant difference between continents as determined by the one-way ANOVA (F (5,436) =5.745, p<0.001). | | | | | |
| Between groups | 193.408 | 5 | 38.682 | 5.745 | <0.001 |
| Within groups | 2935.879 | 436 | 6.734 |  |  |
| Attitude: There was a statistically significant difference between continents as determined by the one-way ANOVA (F (5,368) =6.184, p<0.001). | | | | | |
| Between groups | 149.718 | 5 | 29.944 | 6.184 | <0.001 |
| Within groups | 1781.804 | 368 | 4.842 |  |  |
| Perception: There was a statistically significant difference between continents as determined by the one-way ANOVA (F (5,364) =3.507, p=0.004). | | | | | |
| Between groups | 120.528 | 5 | 24.106 | 3.507 | 0.004 |
| Within groups | 2501.861 | 364 | 6.873 |  |  |
| Practice: There was a statistically significant difference between continents as determined by the one-way ANOVA (F (5,351) =9.521, p<0.001). | | | | | |
| Between groups | 334.016 | 5 | 66.803 | 9.521 | <0.001 |
| Within groups | 2462.645 | 351 | 7.016 |  |  |
| Policy awareness: There was a statistically significant difference between continents as determined by the one-way ANOVA (F (5,374) =3.995, p=0.002) | | | | | |
| Between groups | 799.364 | 5 | 159.873 | 3.995 | 0.002 |
| Within groups | 15046.092 | 376 | 40.016 |  |  |

Table 2: Thematic analysis phase (Braun and Clark, 2006) and researcher’s analysis process

| Thematic analysis phases | Researcher’s analysis process |
| --- | --- |
| Familiarisation with the data | This was started in Qualtrics by developing word cloud from the responses. The Qualtrics file was exported into excel, familiarisation was done while separating the quantitative data from the qualitative data. The excel file was later imported into NVIVO software. Data was read and re-read. |
| Generating initial codes | While familiarising with the extracted data, notes were made by looking at the open-ended questions. We started with ‘harmful environmental nursing practices’ question. We started by generating some codes [in NVIVO] and adding my thoughts to it. We continued to link the various codes under that question’s response [done manually]. We repeated the same process for the remaining questions, which produces lots of initial codes such as ‘*overconsumption of single used items, CPD will help, awareness creation, group autonomy will help, leadership must-not junior staff, No, due to shift pattern and staff shortage, confused priority, issues with curriculum, mandatory training*’.  We started comparing, combining similar codes. We considered the research objectives to guide me in categorising the codes for the whole data set. |
| Searching for themes | This stage was done in word document and researcher’s log book. The codes (in their categories) were entered in tabular form in word document. We began to create initial subthemes of the data which aided in organisation and comprehension of data. Some of the codes were combine into subthemes and themes based on similarity in meaning. For example; combining codes such as ‘*over-consumption of single used items, waste disposal, waste treatment, Intravenous items, and energy use*’ into healthcare carbon footprint sources. |
| Reviewing themes | By checking and looking at the codes, initial themes and subthemes, we re-organised these in a table in Word. We revised each theme and made notes on them. We also made sure the themes had connections its flow to aid report writing clarity. Some of the themes had subthemes [because they had enough information that required distinctiveness in presentation] while other themes were stand alone. |
| Defining and naming themes | Defining and naming themes was performed iteratively from the familiarisation, when we was going through the data and also during the coding stage, we made notes of themes as we identified them. These names where discussed with the research team members to ensure best fit words and enhance comprehension. |
| Producing the report | The findings were produced iteratively from coding, defining themes and making notes throughout the analysis to aid in formulating better understanding of themes. There was some refining, going back and forth exploring the data, and making sure the reader would easily understand without difficulties. There were comparison with research aim and my systematic literature review to make sure the objective of the survey is met. The report was presented in a coherent and logical way with some selected data extracts or individual quotes. |

Table 3: Global hemispheric divide

| **Countries by North and South Divide concept** | **Frequency (n=470*)** | **Percentage (%)** |
| --- | --- | --- |
| Global North | 281 | 59.8% |
| Global South | 189 | 40.2% |

*missing=3
