# Supplementary material for: Climate change and sustainable healthcare practices in nursing: A multi-country exploratory online survey
Source: J Clim Chang Health. 2026 Apr 16;29:100656. doi: 10.1016/j.joclim.2026.100656 (PMC13101775; doi:10.1016/j.joclim.2026.100656)
Supplement: Supplementary file 3 [file mmc3.doc]

**
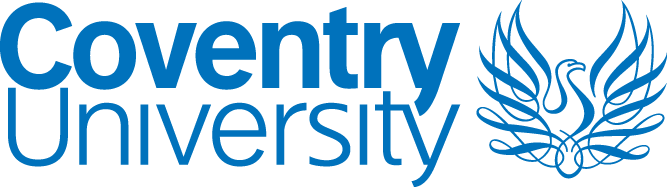
**

**Demographic features**

**Please how do you identify your gender?**

- Man
- Woman
- I identify my gender as:… (please specify)
- Prefer not to say

**Age**

- Below 18 years
- 18-30years
- 31-40years
- 41-50years
- Above 50years

**Education level**

- Certificate in Nursing/midwifery
- Diploma
- Associate Degree
- Bachelor degree
- Master
- Doctor of Nursing Practice
- Professional doctorate in Midwifery
- PhD
- Other (please specify)

**Years of work experience**

- Less than 3 years
- 3-5 years
- 6-10 years
- Greater than 10 years

**What is your level of seniority of your position?**

- Junior
- Middle
- Senior
- Consultant
- Others

**In which country do you practice?** ........................................

**In which hospital unit/department do you currently work? Please tick all that apply.**

- Medical
- Surgical
- Emergency
- Obstetrics & Gynaecology
- Renal
- Respiratory
- Oncology
- Maternity
- Cardiology
- Frailty/ older adults
- Stroke
- Intensive Care Unit
- Theatre
- Paediatrics
- Gastro ward
- Out-Patient Department
- Primary care
- Community Health
- Others (please specify)

**Role**

- Licensed practical nurse (or Band 3, 4 nurse, or Licensed Vocational Nurse)
- Staff nurse
- Shift in-charge/clinical sister
- Clinical lead
- Ward manager
- Advanced practitioner / Specialist
- Matron
- Clinical nurse Educator
- Nurse-midwife
- Research nurse
- Others (please specify)

Climate change refers to the idea that the world’s average temperature has been increasing for the past 50 to 100 years, and may increase more in the future, and the world’s climate may be changing as a result. What do you think: Do you think that climate change is happening?

- Yes
- No
- I don’t know

**Please indicate your level of familiarity with the following statements regarding climate change.**

|  | Not at all familiar | Slightly familiar | Moderately Familiar | Extremely familiar |
| --- | --- | --- | --- | --- |
| Climate change is caused by the increased concentration of carbon compounds in the atmosphere, which are largely generated by human activities. |  |  |  |  |
| Healthcare systems accounts for approximately 5% of total greenhouse gas emissions on the planet. |  |  |  |  |
| Climate change affects the health of human beings and also causes the development of diseases. |  |  |  |  |
| Vulnerable populations such as the very young or old experience more adverse health impacts from climate change. |  |  |  |  |

**Please tell us about your attitude towards climate change. Are you**

|  | Not at all | A little | Moderately | Extremely |
| --- | --- | --- | --- | --- |
| Willing to participate in climate change actions in your organization. |  |  |  |  |
| Interested in global advocacy campaigns on climate change. |  |  |  |  |
| Actively involved in climate change advocacy campaigns or actions. |  |  |  |  |

**Please tell us about your thoughts on climate change and nursing**

|  | Strongly disagree | Disagree | Agree | Strongly agree |
| --- | --- | --- | --- | --- |
| There is a link between climate change and nursing practice. |  |  |  |  |
| Nursing activism could help slow down the progress of climate change. |  |  |  |  |
| There are environmentally friendly practices that could be introduced into nursing practice |  |  |  |  |
| Nurses could influence their employers to introduce environmentally friendly healthcare practices |  |  |  |  |

**Please tell us about your awareness of environmentally friendly healthcare initiatives.**

|  | Not at all aware | Slightly aware | Moderately aware | Extremely aware |
| --- | --- | --- | --- | --- |
| My workplace or institution has | | | | |
| green or net-zero or climate change initiative |  |  |  |  |
| environmental sustainability office or lead |  |  |  |  |
| A nurse on procurement team |  |  |  |  |
| reminders on climate actions posted around the workplace |  |  |  |  |
| transportation scheme geared towards net zero healthcare |  |  |  |  |
| guidelines by the waste bins to ensure proper segregation of healthcare plastic waste |  |  |  |  |

**Please tell us about your workplace mandatory training**.

|  | YES | NO | NOT SURE | Not applicable |
| --- | --- | --- | --- | --- |
| I have had some training at work on environmentally friendly healthcare practices. |  |  |  |  |
| I have experienced awareness raising education at work about climate change and healthcare |  |  |  |  |
| There is a mandatory training course on climate change and nursing practice at work. |  |  |  |  |
| I would be interested in knowing more about how nursing practices and climate actions |  |  |  |  |

To what extent do you agree to the following statements in your workplace?

|  | Strongly disagree | disagree | agree | Strongly agree |
| --- | --- | --- | --- | --- |
| My workplace or organization | | | | |
| makes staff aware of the organization’s values towards the environment |  |  |  |  |
| regularly updates staff on climate change interventions and actions |  |  |  |  |
| makes staff feel welcome to suggest environmentally sustainable actions or ideas |  |  |  |  |
| makes it easy and encourages staff to protect the environment at work |  |  |  |  |
| promotes the use of video or tele-conferencing to avoid business travel where necessary |  |  |  |  |
| mostly operates digitally to reduce paper usage |  |  |  |  |

**Range of environmentally friendly healthcare practices**

How often do you perform the following practices at the workplace?

|  | Rarely | About half the time | Most of the time |
| --- | --- | --- | --- |
| Segregation of healthcare plastic waste |  |  |  |
| Putting off lights and electronic appliances such as monitors when not in use (only when this would not harm patients). |  |  |  |
| Raising questions about procurement of packaged items used in the workplace. |  |  |  |
| Encouraging sustainable ideas (green champion) |  |  |  |
| Travelling to work using shared or public transportation, bike or walking |  |  |  |
| Being water efficient (turning off taps when not in use) |  |  |  |

To what extent do you agree to your use of personal protective equipment, PPE in your workplace?

|  | Strongly disagree | disagree | agree | Strongly agree |
| --- | --- | --- | --- | --- |
| I feel able to decide when and when not to use PPE when providing nursing care. |  |  |  |  |
| I always wear PPE when providing nursing care because it is expected. |  |  |  |  |
| When there is no risk of infection, I make a decision not to use PPE to reduce plastic waste. |  |  |  |  |

Please do you know what term 'Net-Zero Healthcare' means?

Yes No Not sure

**In your opinion, what are the main barriers to practicing environmentally friendly healthcare; Tick the top 5 that apply*.***

Busy shift/ Work overload

Inadequate implementation of environmental policies

I do not know enough about climate change

I do not know what to do

I believed my role should only focused on patient need

Less consciousness of the environmental care

It is not my duty to reduce the carbon footprint of my workplace

My climate change actions have very little to offer

Lack of organisation support

Lack of autonomy in decision making

Unnecessary procurement

I am not confident to act

Fear of getting infections

Others (please specify)

Some nursing practices have a harmful impact on the environment. *If you are agree, can you list some of these nursing practices?*

Is it possible to reduce plastic waste where you work?

Yes No

*If yes, please what aspects could you suggest?*

Do you feel you have the autonomy to influence climate change actions at your workplace? Please share your views...............

Do you feel it is your role to try to influence climate change? Please share your views

If you are a nurse educator/lecturer/ nurse leader, is there any sustainable actions or barriers to comment? Please share your views
