# Supplementary material for: Climate change and sustainable healthcare practices in nursing: A multi-country exploratory online survey
Source: J Clim Chang Health. 2026 Apr 16;29:100656. doi: 10.1016/j.joclim.2026.100656 (PMC13101775; doi:10.1016/j.joclim.2026.100656)
Supplement: Supplementary file 1 [file mmc1.docx]

Response to editor-survey manuscript

Dear Editor,

Thank you very much for the opportunity, review and feedback.

References (1, 2, 3, 5, 8, 25) have been edited to closely match the standard reference format for JoCCH submissions.

| Editor comments | |
| --- | --- |
| Writing "&" or "and" at the end of author lists is unnecessary; please remove these. Additionally, please remove the comma before the year of publication for all articles. This has been amended. | |
| 1 cite as book | Lemery, J., Knowlton, K., Sorensen, C. Global climate change and human health: from science to practice. 2nd ed. San Francisco: John Wiley & Sons; 2021. |
| 2 remove all abbreviations and cite as report | Intergovernmental Panel on Climate Change. Climate Change 2013: The Physical Science Basis. Contribution of Working Group I to the Fifth Assessment Report of the Intergovernmental Panel on Climate Change. Cambridge, United Kingdom and New York, NY, US: Cambridge University Press; 2013. 1535 pp. <https://www.ipcc.ch/site/assets/uploads/2018/02/WG1AR5_all_final.pdf> |
| 3 cite as book | Romm, J. Climate change: What everyone needs to know. 3rd ed. New York: Oxford University Press; 2022. |
| 5 cite as article | Kahn, ME, Ouazad, A, Yönder, E. Adaptation using financial markets: Climate risk diversification through securitization. National Bureau of Economic Research. 2024; (No. w32244): 2-10. <https://www.nber.org/papers/w32244> |
| 8 cite as book chapter | Nieto-Cerezo, O. Refining NHS Climate Change Adaptation Plans: Central Manchester University Hospitals Foundation Trust (CMFT) Case Study. In: Leal Filho, W., editors. Innovation in Climate Change Adaptation. Climate Change Management. Switzerland: Springer International Publishing; 2016. pp. 225-245. <https://doi.org/10.1007/978-3-319-25814-0_16> |
| 25 cite as computer software | QSR International Pty Ltd. NVivo qualitative data analysis software. Melbourne: Lumivero; 2018. Internet. cited 2023 Nov 6 Available from: <https://www.qsrinternational.com/nvivo-qualitative-data-analysis-software/home> |

Regards

Ebenezer Akore Yeboah
